# Patient treatment cost of oral diseases in Ghana

**DOI:** 10.4314/gmj.v56i3.7

**Published:** 2022-09

**Authors:** Seli Y Deh, Justice Nonvignon, Moses Aikins, Samuel A Agyemang, Genevieve C Aryeetey

**Affiliations:** 1 Tema Metropolitan Health Directorate, Ghana Health Service, Private Mail Bag, Tema, Ghana; 2 School of Public Health, College of Health Sciences, University of Ghana, P.O. Box L.G. 13, Legon, Ghana; 3 Health Economics, Systems and Policy Research Group, University of Ghana, Legon- Accra, Ghana

**Keywords:** Oral diseases, direct cost, indirect cost, sensitivity analysis, treatment, Ghana

## Abstract

**Objectives:**

To estimate patient treatment cost of oral diseases in Ghana

**Design:**

A cross-sectional study design using cost-of-illness analysis was employed

**Setting:**

The study was conducted at the dental unit of the University of Ghana Hospital, Legon

**Participants:**

About 185 patients attending the dental unit of the hospital were selected

**Interventions:**

None

**Main outcome measures:**

Direct medical and non-medical costs, indirect costs, and intangible costs of treatment of oral conditions

**Results:**

The estimated average cost of treatment for oral diseases was US$ 35.75. The total cost was US$ 6,614.11, with the direct and indirect costs constituting 94.5% and 5.5%, respectively of the total cost. Direct medical costs constituted 86.9%, while direct non-medical costs constituted 13.1% of the total direct cost. The richer socio-economic group had the highest cost per quintile, with a mean of US$ 46.69. The intangible cost described was highest for pain (47.1%), followed by difficulty in eating (40.8%) and sleeping (34.6%) for both men and women.

**Conclusion:**

The costs of oral diseases are huge and cannot be overlooked. Oral diseases also pose significant productivity losses to patients.

**Funding:**

None declared

## Introduction

Oral health is essential to the overall well-being of an individual. Poor oral health results from poor oral hygiene and other oral health-related problems. Some oral diseases include oral infections, trauma, injuries, hereditary lesions, and oral cancer. Currently, it is estimated that about 3.9 billion people are affected by oral diseases worldwide. Periodontal disease and dental caries, the two commonest dental diseases, affect 20% and 90% of the world's population. The burden of oral disease has risen by 21% in the last 20 years.^1^

Oral diseases are common in both developed and developing countries. In the last 30 years, there have been improvements in the prevalence of dental caries in Europe.^2^ In developing countries, the prevalence of the disease is still increasing despite many interventions undertaken to reduce the incidence of the disease. Studies have reported caries prevalence of 72% among rural Indian adolescents and 51% among Pakistani preschoolers.^3^,^4^

North African countries like Egypt and Tunisia have prevalence rates of about 70% among preschoolers and 43% among university students, respectively.^5^,^6^

According to the 2006 World Health Survey - a survey of adults from 72 selected countries - adults in Africa and Southeast Asia experienced a lower loss of their natural teeth (edentulism) than their American and European counterparts. Again, the prevalence of edentulism was high (35%) in upper-middle-income countries and about 10% in lower-income countries.^7^,^8^ Various studies have reported dental carries as the commonest dental condition among children with a high prevalence, around 44% among adolescents. The presence of plaque, calculus and gingivitis are common among adolescents.^9^–^12^ Studies have also found an association between periodontal disease and cardiovascular and other diseases.^13^–^16^ A study reported lower medical costs and hospitalisations in patients with either type II diabetes, cerebral vascular disease or coronary artery disease following periodontal treatments compared to untreated controls.^17^

Although few studies on the overall disease burden in Africa, the World Health Organization reports that Eastern, Central and sub-Saharan Africa bear the highest burden of oral diseases.^18^ Studies conducted in Nigeria and Ghana reported caries prevalence of 60% and 55%, respectively.^19^,^20^ Korle-Bu Teaching Hospital in Ghana recorded an increase of 75% in dental infections from 2011 to 2012.^21^

The World Health Organization identifies oral diseases as the most expensive to treat.^18^ Oral health care accounts for between 5% and 10% of total health care expenditures in industrialised countries.^22^ Countries within the European Union spent €54 billion and €79 billion on dental healthcare in 2004 and 2009. This amount is estimated to be €93 billion in 2020.^2^ Further, in the United States, the total dental expenditure for children aged 5–17 years amounted to $20 billion in 2009, while total expenditure on dental services amounted to $110.9 billion in 2012. Again, out-of-pocket spending for dental services increased by 3.0% in 2012.^23^,^24^

The situation is different in Low- and Middle-Income Countries (LMICs), with unmet population needs for oral health care. For instance, Tanzania's oral healthcare budget is inadequate to meet the increasing oral health needs of the population. Also, individual households in Kenya finance oral health care mainly from out-of-pocket payments.^25^,^26^ The Ghanaian health sector has undergone major policy changes during the past decades. These changes resulted in the restructuring of the entire health sector.^27^ Ghana's current Universal Health Coverage (UHC) journey began in 2003 when the National Health Insurance Act 650 was passed by parliament.^28^ About 60 % of Ghana's population consists of Social Security and National Insurance Trust (SSNIT) contributors and pensioners, those regarded as too poor to afford the insurance premium, including Livelihood Empowerment Against Poverty (LEAP) beneficiaries (indigents), children less than 18 years of age, persons aged above 70 and pregnant women are excluded from paying out of pocket premiums.^29^ Recently, enrolment in the National Health Insurance Scheme (NHIS) has stagnated, with lower socio-economic status and large household sizes contributing to the inability to afford full insurance.^30^ The basic dental treatments in Ghana are covered by the NHIS and other private insurance providers, but dental patients are compelled to make out-of-pocket payments for some treatments not covered by the NHIS. With limited insurance coverage, out-of-pocket spending for health, including dental care, becomes common. This may result in health ‘shocks’, increasing the tendency to impoverish vulnerable households.^31^,^32^

Given that treatment cost for dental diseases has been regarded as high, studies on the burden of the disease on patients, particularly in developing countries such as Ghana, is limited. Hence, this study aims to estimate the burden of oral diseases on patients by estimating direct and indirect costs associated with treating the disease.

## Methods

### Study design and setting

The study was a cross-sectional cost-of-illness (COI) analysis conducted at the University of Ghana Hospital, a quasi-government hospital in Accra, Ghana. The hospital provides healthcare to the University of Ghana community, including employees and students. It also draws patients from neighbouring communities. It, however, does not accept patients insured under the NHIS. Hence all other patients apart from employees and students of the University are treated as private patients. Ethical approval for the study was obtained from the Ghana Health Service Ethics Review Board with the Ethics Approval Certificate Identification Number (GHS-ERC48/02/15). The purpose of the study was also explained to the respondents, and their written consent was obtained.

### Study population, sample size and sampling

The study population consisted of dental patients who attended the Dental Clinic of the University of Ghana Hospital, Legon. The prevalence of oral disease in adults in Ghana is undocumented. Therefore, assuming a prevalence of 50% for oral diseases in adults, the sample size (n) was calculated using the Cochrane Formula and the finite population correction factor.^33^

The study's population (finite population) for the period was around 300 patients per month. Therefore, using the finite population correction factor gave a sample size of 168. Assuming a 10% non-response rate, it gave 17 additional patients. Hence the final sample size was 185. Thus, a random sampling technique was used to identify 185 study participants accessing dental care over the 30-day data collection period. Inclusion criteria were patients 18 years and above receiving treatment at the dental department, while those who came for consultation and review at the same department were excluded.

The attendance list for all patients visiting the facility for the day was obtained. This list was made available at the outpatient department's vital signs unit. A list of patients who met the inclusion criteria, i.e. those with real evidence of dental disease and receiving treatment, was extracted from the attendance list. Participants were randomly selected using a ballot system, and interviews were administered to those who gave their consent. This procedure was repeated every day until the desired sample size was achieved.

### Data Collection and tools

Data were collected over one month in May 2015. Measures taken to ensure data reliability included training research assistants for data collection, pretesting the questionnaire, editing the completed questionnaire, and designing an appropriate screen for data entry. An individual with requisite background in dental health care and who could speak two common local dialects (Twi and Ga) was recruited and trained to serve as a research assistant for the study.

The questionnaire was pretested at the dental unit of the Greater Accra Regional Hospital, Accra.

The pretest was to identify ambiguity and other difficulties participants may encounter in responding to the questions. Data were collected from patients who agreed to participate after obtaining informed consent. The background characteristics of respondents included socio-demographic characteristics, asset ownership (i.e. wall clock, television, fridge/freezer, washing machine, home theatre, bed/table/chair, house, radio, computer/laptop, VCD/DVD player, sewing machine, cabinet/ cupboard, motorcycle/scooter, generator, plot of land), type of dental condition and type of treatment received. Direct cost variables comprised medical costs (consultation, diagnostics, treatment, and medication) and non-medical costs (travel, food, and miscellaneous expenses). Indirect costs covered productivity days lost, travel time, and waiting time. All the costs estimated in this study were costs for which the participants made direct cash payments. A description of the study variables is presented in Table 1.

**Table 1:** Description of study variables

Cost type	Cost component	Estimation undertaken
Direct cost Medical costs:	*Registration & consultation:*	This was the summation of the costs of registration and consultation of the patients during the visit.
	*Diagnostics:*	This is the summation of the cost of diagnostic tests of the patients during the visit. Diagnosis were confirmed from patient's folders.
	*Treatment:*	This is the summation of the costs of the treatments of the patients during the visit.
	*Medication:*	This is the summation of the medications prescribed for the patients during the visit.
	*Total medical cost:*	This is the summation of the total costs of registration, consultation, diagnostics, treatment and medication for received by the patients during the visit.
	Non-medical costs:	
	*Travel:*	This is the summation of all travel costs incurred by the patient to and from home to the Dental Clinic during the visit.
	*Food:*	This is the summation of all food costs incurred by the patients during the visit.
	*Miscellaneous:*	This is the summation of all miscellaneous costs incurred by the patients (i.e., telephone calls or other pain reliving agents) related to their dental diseases.
	*Total direct non-medical* *cost:*	This is the summation of all travel costs, food costs and miscellaneous expenses incurred by the patients due to their dental diseases.
Indirect costs	*Total travel time:*	This is the summation of the time spent (hours) travelling to and from home to the Dental Clinic.
	*Valued travel time:*	This was estimated by multiplying the total travel time spent by patients who are employed by the hourly rate of the daily minimum wage.
	*Total waiting and treatment* *time:*	This is the summation of the times spent (hours) on waiting and treatment at the hospital.
	*Productivity days lost:*	This is the summation of the total number of days lost by patients who are employed in seeking dental care.
	*Valued productivity days* *lost:*	This was estimated by multiplying the total number of days lost by patients employed by the daily minimum wage.
	*School days lost:*	This is the summation of the number of days lost by student patients seeking dental care.
	*Total indirect cost:*	This is the summation of valued travel time, waiting and treatment time and productivity days lost by dental patients.

### Data Analysis

#### Descriptive analysis

Some socio-demographic characteristics of the study participants were analysed, including age, sex, marital status, education, asset ownership, and duration of the dental condition, among others. Also, the chi-square test, t-test, and statistical significance in cost difference were determined using Kruskal-Wallis and means for demographic and cost variables.

#### Cost Analysis

Costs were analysed from the patient's perspective. The direct cost was estimated as costs incurred by the patients for treatment. It included both medical and non-medical costs. Total indirect cost was estimated using the human capital approach (HCA), which measures output losses by lost earnings.^34^ Productivity losses were estimated by calculating the total work hours lost and total lost earnings using the minimum daily wage in Ghana at the time of the study. The cost estimations were carried out in local currency and later converted to U.S. Dollar (US$) equivalent using the Bank of Ghana's annual average foreign exchange rate for the US$ in 2015. The cost of productive days lost was calculated for employed patients only. Days lost by the unemployed and students were not included. Intangible costs in this study were not estimated but described using a five Likert scale item. The scale items assessed the patients' ratings for pain, difficulty with chewing, difficulty with speaking, difficulty with smiling and avoiding the company of others due to dental disease.

#### Sensitivity analysis

A sensitivity analysis was performed to assess the robustness of the cost estimates. This was done by varying the medication cost and the minimum wage by 5%, 10% and 25%.

### Estimation of socioeconomic status

Respondent's socioeconomic status (SES) was estimated to rank them into wealth quintiles. Various household asset ownership, as reported by the respondent, was used as a proxy for wealth. Principal components analysis (PCA) was then used to estimate an SES score. Respondents were ranked into wealth quintiles based on their SES score.^32^,^35^ All analyses were conducted using Microsoft Excel 2007^36^ and STATA version 12.^37^

## Results

### Background characteristics of respondents

The background characteristics of respondents are in Table 2. Table 2 shows that about 45% were males and 55% were females. The highest age category was those between 20–29 years (57%). Most respondents had some tertiary education (80%), and over 80% were not married. Also, 39.5% of the respondents have had their condition for less than a week, while 25.4% from a week to a month and 35.1% have had their condition from one month to a year.

**Table 2:** Background characteristics of respondents

Characteristics	Number (%/days)	Average cost (US$)	P-value
**Sex**+:			< 0.001***
**Male**	82 (44.3)	39.22 (54.3%)	
**Female**	103 (55.7)	33.07 (45.7%)	
**Age:**			
**<20**	24 (12.9)		
**20–29**	105 (56.8)		
**30–39**	21 (11.4)		
**40–49**	14 (7.6)		
**50–59**	10 (5.4)		
**60+**	11 (5.9)		
**Marital Status+:**			< 0.001***
**Married**	34 (18.4)	44.25 (57.0%)	
**Not** **married**	151 (81.7)	33.38 (43.0%)	
**Educational Status**Δ:			0.809
**No education** **/Primary**	9 (4.9)	30.24 (21.7%)	
**Middle** **school/JHS**	10 (5.4)	29.80 (21.4%)	
	18 (9.7)	43.88 (31.5%)	
**Tertiary**	148 (80.0)	35.50 (25.5%)	
**Employment Status**Δ:			< 0.001***
**Employed**	67 (36.2)	40.11 (29.0%)	
**Students**	100 (54.1)	26.34 (19.1%)	
**Unemployed**	18 (9.7)	71.77 (51.9%)	
**Productive days lost**			
**Employed**	67 (124 days)		
**Students**	100 (136days)		
**Duration of condition:**			
**<Week**	73 (39.5)		
**<Month**	47 (25.4)		
**<Year**	65 (35.2)		

Δ ANOVA

+ t-test

* p < 0.05

** p < 0.01

*** p < 0.001 significant levels

### Oral conditions of respondents and type of treatment received

In Table 3, toothache accounted for 54.6% of oral conditions reported by the respondents at the dental clinic. This was followed by gum disease, reported by 27% of the respondents. Swollen jaw (dentoalveolar abscess) was reported by about 4% of respondents. Broken teeth, mouth sores and other dental conditions, including tooth sensitivity, pericoronitis and impacted lower third molar, were also reported. Treatment received by respondents included extraction (21.6%), filling (8%) and scaling and polishing (21.1%). There was no statistically significant difference in total cost for respondents reporting different conditions, as reported by the Kruskal-Wallis test (p=0.433). Similarly, there was no statistically significant difference in average cost and the various treatment categories (p=0.831).

**Table 3:** Average cost of oral conditions and type of treatment

	Number (%)	Average cost (US$)(SD)	p-value†
**Oral condition:**			0.433
**Bad Breath**	3 (1.6)	30.81 (1.22)	
**Broken Tooth**	15 (8.1)	64.48 (62.09)	
**Gum Disease**	50 (27.0)	34.9 (10.84)	
**Mouth sores**	5 (2.7)	26.94 (5.94)	
**Swollen Jaw**	8 (4.3)	27.41 (9.14)	
**Toothache**	101 (54.6)	31.09 (52.42)	
**Other** a	3 (1.6)	104.95 (6.49)	
**ype of treatment:**			0.831
**Cleaning**	39 (21.1)	35.13 (37.76)	
**Dentures**	4 (2.2)	24.52 (8.36)	
**Extraction**	40 (21.6)	38.47 (66.31)	
**Filling**	15 (8.1)	55.45 (14.21)	
**Medication**	81 (43.8)	31.55 (35.76)	
**Other** aa	6 (3.2)	36.62 (40.92)	

a Other dental conditions include dentoalveolar abscess, tooth sensitivity and pericoronitis

aa Other treatments include root canal treatment and excision of epulis

*p < 0.05, **p < 0.01, ***p < 0.001 significant levels

† Kruskal-Wallis test used to determine statistical significance in mean difference of more than two categories

### Direct and indirect cost

The average cost of treatment was estimated at US$ 35.75, with the total cost for the study sample being US$ 6,614.11. About 94.5% of total costs were direct costs (US$ 6,248.59), with the remaining 5.5% being indirect costs (US$ 365.52).

The average direct and indirect costs were US$ 33.78 and US$ 1.98, respectively. The major cost driver was direct medical cost (82.1%), of which treatment and consultation were the two major contributors. The direct and indirect costs distribution and cost profile of oral diseases is shown in Table 4.

**Table 4:** Cost profile for treatment of oral diseases

Cost component	Items	Cost (US$)*	Average Cost (US$)*	SD	Cost profile (%)
**Direct costs**					
**Medical costs:**	Consultation	1,774.94	9.59	4.4	26.8
	Diagnostics	298.05	1.61	3.7	4.5
	Treatment	2,435.52	13.16	37.8	36.8
	Medication	918.49	4.96	4.1	13.9
	**Sub-total**	5,427.01	29.33		82.1
**Non-medical costs:**	Travel	510.61	2.76	7.9	7.7
	Food	233.24	1.26	1.61	3.5
	Miscellaneous	77.74	0.42	1.2	1.2
	**Sub-total**	821.58	4.44		12.4
	**Total direct cost**	6,248.59	33.78		94.5
**Indirect costs**					
	Valued productive days lost	211.19	1.14	4.11	3.2
	Valued travelling time	76.39	0.41	0.39	1.2
	Valued waiting time	77.94	0.42	0.31	1.2
	**Total indirect**	**365.52**	1.98		5.5
	**Total cost**	**6,614.11**	35.75		100

* Daily interbank US$ forex rate of US$1.00 = GHS 4.11 (10^th^ June, 2015). *The national minimum wage per day (GHS 7.00) as at June, 2015 was used to value productivity days and time lost to patients.

Patients lost a total of 260 productive days from being absent from either school or work due to dental diseases. The employed reported a total of 124 days (mean 1.9 days) lost, while the unemployed, including students, reported total days lost of 136 (mean 1.3 days). On average, each patient lost 1.6 days from dental disease treatment. Our study also showed a significant difference in mean cost for oral treatment by gender, marital status and employment status. For example, females spent around US$33 for oral treatment compared to US$39 for males (p=0.000). Similarly, those who were married paid around US$44 compared to US$34 paid by those who were not married (p=0.001).

### Distribution of direct cost by socio-economic quintile

Table 5 revealed that the respondents in different socio-economic groups incurred different costs. The respondents in the 4th wealth quintile incurred the highest direct cost per quintile, which amounted to US$ 1,680.80 and those in the 5th wealth quintile incurred the lowest cost, which amounted to US$ 1,014.70. Nonetheless, there was no statistically significant difference in direct cost for participants of different socioeconomic statuses, as reported by the Kruskal-Wallis test (p=0.192).

**Table 5:** Direct cost of oral diseases by socioeconomic status

		Direct Cost	Average direct cost	Proportion of cost burden to overall average cost (%)	p-value†
Wealth quintile	Number	US$	(US$)*		
					0.192
**1^st^ Quintile**	37	1,039.22	28.09	78 .6 (18.82)	
**2^nd^ Quintile**	37	1,200.44	32.44	90.7 (39.13)	
**3^rd^ Quintile**	38	1,313.43	34.56	96.7 (36.99)	
**4^th^ Quintile**	36	1,680.80	46.69	130.6 (76.55)	
**5^th^ Quintile**	37	1,014.70	27.42	76.7 (11.98)	
**Total**	**185**	**6,248.59**			

Daily interbank US$ forex rate on 10^th^ June, 2015 used was GHS 4.11

* p < 0.05

**p < 0.01, ***p < 0.001 significant levels

† Kruskal-Wallis test used to determine statistical significance in mean difference of more than two categories Direct and indirect cost

### Distribution of direct cost by socio-economic quintile

Table 5 revealed that the respondents in different socio-economic groups incurred different costs. The respondents in the 4th wealth quintile incurred the highest direct cost per quintile, which amounted to US$ 1,680.80 and those in the 5th wealth quintile incurred the lowest cost, which amounted to US$ 1,014.70. Nonetheless, there was no statistically significant difference in direct cost for participants of different socioeconomic statuses, as reported by the Kruskal-Wallis test (p=0.192).

### Intangible cost expressions

Males mostly complained of pain, difficulty with smiling and sleeping as well as difficulty with eating different food varieties due to pain or discomfort. Whilst females complained of difficulty speaking, eating and avoiding the company of others.

### Sensitivity Analysis

Sensitivity analysis showed that the multi-variation of medication cost and wage rate produced the highest percentage change in the total cost (i.e., 0.8%, 1.5% and 3.8%). This makes the total cost most sensitive to the multi-variation cost of medication and wage rate. The proportions of the direct and indirect costs were not sensitive to the 5% multi-variation of the cost of medication and wage rate. They were, however, sensitive to 10% and 25% multi-variations. Also, the proportions of the indirect costs were more sensitive to the multi-variations than the proportions of the direct cost and the proportions of the direct cost decreased as the multi-variations increased however, since the changes produced by the variations were close to the initial proportions of the direct cost (94.5%) and indirect cost (5.5%) it shows that the cost estimates are robust (Table 6

**Table 6:** Sensitivity analysis of total cost of oral diseases

Scenario	Cost component	Percentage change in parameter		Percentage change in total cost	Proportions of total cost		Percentage change in proportions of cost	
			(US$)***		Direct	Indirect	Direct	Indirect
**Base scenario**		**0**	**6,614.11**	**0**	**94.5**	**5.5**	**0**	**0**
**Variation** *	Medication	5	6,660.04	0.7	94.5	5.5	0.0	0.0
		10	6,705.96	1.4	94.5	5.5	0.0	0.0
		25	6,843.73	3.5	94.7	5.3	0.2	0.0
**Variation** *	Wage rate**	5	6,617.90	0.1	94.4	5.6	-0.1	1.8
		10	6,621.80	0.1	94.4	5.6	-0.1	1.8
		25	6,635.77	0.3	94.2	5.8	-0.3	5.5
**Multi-variation**	Medication and wage rate	5	6,663.83	0.8	94.5	5.5	0.0	0.0
		10	6,713.54	1.5	94.4	5.6	-0.1	1.8
		25	6,865.39	3.8	94.4	5.6	-0.1	1.8

* The cost of medication and the wage rate was varied by 5%, 10% and 25% increment respectively.

** The national minimum wage per day (GHS 7) as at June, 2015 was used to value productivity days lost to patients.

*** Daily interbank US$ forex rate on 10^th^ June, 2015 used was GHS 4.11.

## Discussion

This study estimated the direct and indirect costs of treatment for oral diseases in a quasi-government facility in Ghana. The study revealed that the total cost of oral diseases was US$ 6,614.11, with an average of US$ 35.75 per treatment. The direct and indirect costs constituted 94.5% and 5.5% of the total cost profile, respectively. Medical and non-medical costs constituted 86.9% and 13.1% of the direct costs, respectively.

The study showed that direct cost constituted the highest proportion of the overall cost and is similar to findings in studies in South East Asia, where medical costs constituted a greater portion of the direct cost, indicating that treatment costs of oral diseases are high and can put an appreciable burden on households in LMICs.^31^,^32^ Again, treatment costs of oral diseases can run into millions of dollars from the societal perspective, where costs are borne by patients, health care providers, health insurance companies and other stakeholders in healthcare.^38^,^39^

High treatment costs arise due to curative rather than preventive oral treatments, and findings in this study and similar studies in other LMICs indicate that many patients (80%) require curative oral treatment. The cost of oral care may prevent patients from seeking preventive or early treatment resulting in a worsening dental problem. Those who do seek treatment have to pay out of pocket. The unavailability of a comprehensive national oral health preventive programme to provide preventive oral health care can result in high treatment costs. Given that the average treatment cost in this study was US$ 35.75 and the current daily minimum wage is US$1.70, Oral diseases can somewhat result in catastrophic spending for some relatively poor households.^36^

The study findings showed a minimum contribution of the indirect costs (5.5%) to the overall cost profile. Even though the study did not estimate the productive days lost in recuperating from the treatment, it estimated productive days lost in seeking care due to the condition. This was around 260 days in total and an average of 1.6 days. Most oral diseases, except for oral cancers and a few others, are not chronic therefore, patients do not need prolonged stay in the hospital and thus may not lose many productive days. Studies in Australia, Canada and the United States estimated about 2 to 3 productive hours lost. This may be due to the availability of many dental facilities in developed countries. In contrast, fewer dental facilities in LMICs lead to reduced access and increased waiting time at the dental facilities. Again, the estimated lost hours may seem small or even insignificant however, these productive hours could run into millions of hours cumulatively, with over a billion dollars lost to productivity.^22^,^34^,^35^,^40^

This study identified pain as the commonest symptom reported by the patients. Some oral diseases are not associated with pain however other oral diseases can cause excruciating pain. In this study, close to 50% of respondents reported having pain associated with their condition. Other studies have reported similar outcomes.^41^–^43^

The psychosocial effect of oral diseases manifests in many forms and affects people differently. The magnitude of this effect may be determined by the individual's self-awareness and the value the society or community and the individual place on oral health. Dental aesthetics seem to be the most revered value, and oral diseases that result in tooth loss, discolouration or mal-alignment of teeth, especially the anterior teeth, affect the patient's ability to socialise. The patients, therefore, have difficulty interacting with people. Other oral diseases which result in bad breath led to poor self-confidence, avoidance behaviours, isolation and depression in certain instances.^44^,^45^

The final marker of most oral diseases is tooth loss. Depending on its severity, it may cause some discomfort and challenges with chewing and speaking, thereby producing changes in speech quality.^46^,^47^ These challenges are usually manifested in patients with multiple tooth loss. However, some patients develop some form of coping mechanisms or acquire dental prostheses to enable them to overcome such challenges.^48^

Some issues are important in the interpretation of the results of this study. First, the study was conducted in a quasi-government institution that did not have a maxillofacial unit, which usually treats chronic oral diseases like oral cancers and tumours requiring hospitalization and may significantly increase the number of productive days lost. Second, since the facility does not accept health insurance, we could not estimate issues regarding the distribution of co-payments of burden by insurance status. Again, costs in the study were estimated only from the patient's perspective; therefore, estimating societal costs in future studies will further enhance knowledge in the economic costs of oral diseases. Finally, though the study sample may not be very representative of the entire Ghanaian population, the unit cost estimates derived could represent those of populations in similar settings, such as University hospitals in urban settings across the country

## Conclusion

The costs of oral diseases are high and cannot be overlooked. High treatment costs may lead to catastrophic health expenditures by poor households. Oral diseases cause productivity losses and have a psychosocial effect on patients.

Oral health intervention programs should focus more on preventive than curative care. Also, pro-poor oral health policies should be formulated to prevent oral diseases, improve oral health and reduce the economic costs associated with oral diseases.

## Figures and Tables

**Figure 1:**
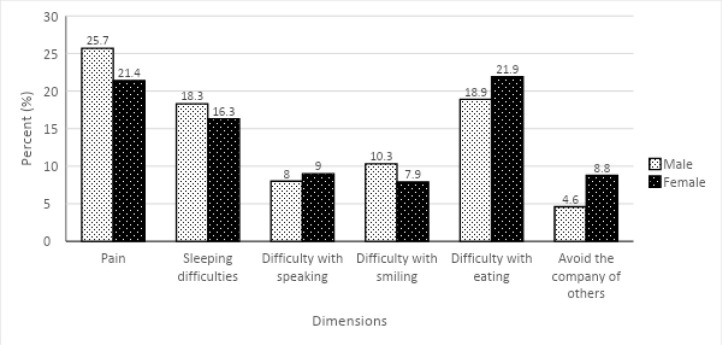
Dental patients' intangible costs expressions
